# Fear of Darkness, the Full Moon and the Nocturnal Ecology of African Lions

**DOI:** 10.1371/journal.pone.0022285

**Published:** 2011-07-20

**Authors:** Craig Packer, Alexandra Swanson, Dennis Ikanda, Hadas Kushnir

**Affiliations:** 1 Department of Ecology, Evolution and Behavior, University of Minnesota, St. Paul, Minnesota, United States of America; 2 Tanzanian Wildlife Research Institute, Arusha, Tanzania; University of Bristol, United Kingdom

## Abstract

Nocturnal carnivores are widely believed to have played an important role in human evolution, driving the need for night-time shelter, the control of fire and our innate fear of darkness. However, no empirical data are available on the effects of darkness on the risks of predation in humans. We performed an extensive analysis of predatory behavior across the lunar cycle on the largest dataset of lion attacks ever assembled and found that African lions are as sensitive to moonlight when hunting humans as when hunting herbivores and that lions are most dangerous to humans when the moon is faint or below the horizon. At night, people are most active between dusk and 10:00 pm, thus most lion attacks occur in the first weeks following the full moon (when the moon rises at least an hour after sunset). Consequently, the full moon is a reliable indicator of impending danger, perhaps helping to explain why the full moon has been the subject of so many myths and misconceptions.

## Introduction

Attacks by man-eating predators commonly occur at night [1]–[5], and nocturnal species typically alter their behavior according to levels of moonlight [6]–[11]. Our innate fear of darkness has long been considered an adaptation to the risk of nocturnal predation [e.g. ref. 12], yet the bright nights of the full moon are associated with widespread superstitions and persistent fallacies about human pathology [13]–[15]. To test whether these perceptions might reflect evolutionary responses to the dangers of nocturnal predation, we measured the food intake of African lions across the lunar cycle and tested whether moonlight affects the timing of lion attacks on humans in southern Tanzania where over 1000 people were attacked between 1988 and 2009.

## Materials and Methods

**Figure 1:**
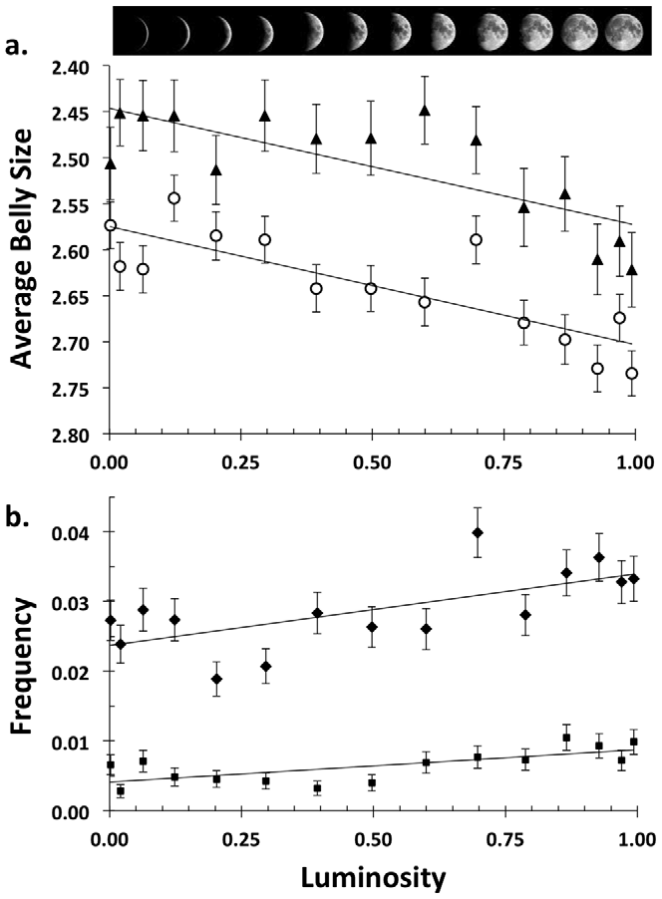
Moonlight and lion behavior in the Serengeti and Ngorongoro Crater. Luminosity is the average proportion of the moon's surface illuminated on that night; vertical lines indicate standard errors. **A** Females (circles) had larger belly sizes and hence consumed more meat on days nearest the new moon (P<0.0001, n = 7,683 sightings); males (triangles) showed a similar pattern (P<0.0001, n = 3,827 sightings). **B.** Lions were more likely to make a daytime kill (diamonds) or scavenge (squares) on days nearest to the full moon (P<0.0001 for both). Regression lines are based on the raw data for each respective measure and are only presented to provide a visual guide to the overall trends; statistical analyses are presented in Tables S1, S2, S3, S4, S5, S6.

Retrospective data on the lunar cycle are available from the US Naval Oceanography website, www.usno.navy.mil/USNO/astronomical-applications/data-services/rs-one-year-world. Serengeti and Ngorongoro lions have been studied continuously since 1966 [16]–[17]; at least one adult female has been fitted with a radio collar in each Serengeti pride since 1984. Beginning in 1978, belly size has been consistently recorded on a scale from 1.0 to 5.0 at 0.25 increments, with 1.0 being the fattest [18]. “Average belly size” is the mean of all females ≥3 yrs of age in each group, using only one sighting per group per day. Statistical analyses of the effects of lunar cycle on belly size (Fig. 1a, Tables S1, S2 & S3) used linear mixed model (function *lmer*, library *lme4*)) in R [19] with random effects of pride and year to control for pseudoreplication; a linear mixed model could be used because belly size is a continuous variable and group averages vary continuously. Behavioral records dating from 1966 specify whether carcasses were obtained by predation or scavenging. Effects of moon phase on carcass acquisition (Fig. 1b, Tables S4, S5 & S6) were tested using a generalized linear model (function *glm*) with Poisson distribution. All results reported in Fig. 1 control for season (wet vs. dry), habitat (Serengeti plains vs. Serengeti woodlands vs. Crater) and season/habitat interactions; details are provided in the Supporting Information.

Records of lion attacks on humans are available from the Tanzanian Government dating back to 1988 [3], [5]. Follow-up visits were made to over 500 attack sites by DI and HK in Kilwa, Kisarawe, Lindi, Liwale, Manyoni, Mkuranga, Mtwara, Newala, Ruangwa, Rufiji, Singida and Ulanga Districts; survivors and victims' families were interviewed to confirm details of the attacks and to collect additional information [5]. Analyses exclude all attacks that occurred during retaliatory lion hunts, leaving data on 474 victims from 450 attack events. All times are +3 hrs GMT; all probabilities are two-tailed. Expected values for Kolmogorov-Smirnov tests are based on the assumption that, for each time of day, attacks were equally likely to have been reported across all days of the lunar cycle. All results presented here were based on the number of victims because 432 of the 450 attack events involved a single victim and the distribution of 2–4 victim attacks closely followed the binomial distribution. However, if the analysis is restricted to attack events, most findings remain unchanged though variation across the lunar cycle is no longer statistically significant at 21:00 or 00:00.

## Results

Lions in Serengeti and Ngorongoro Crater enjoy higher food intake during moonless nights, as measured by the belly sizes of adult females and adult males, which are significantly larger on days closest to the new moon (Fig. 1a). Although males have higher average belly size than females, the slope with luminosity was virtually identical for the two sexes (Tables S2 & S3). Extensive studies of nocturnal foraging in South Africa and Uganda have shown that lion hunting success is highest on dark nights [6], [9], and we similarly found lions feeding on a higher proportion of mornings near the new moon (P<0.01, n = 2,975 carcasses; Table S4); carcasses can persist for hours, thus morning observations largely reflect prey acquisition before dawn. Lions try to compensate for lower nocturnal food intake around the full moon by killing and scavenging more during daylight hours (Fig. 1b; Tables S5 & S6). However, these daytime responses are inadequate to overcome their low nocturnal food intake around the full moon: a lion's belly size reflects its total food intake. Note that food acquisition and belly sizes are similar during the first and third quarters of the lunar cycle; the effect of moonlight is the same regardless of whether the moon is waxing or waning.

**Figure 2:**
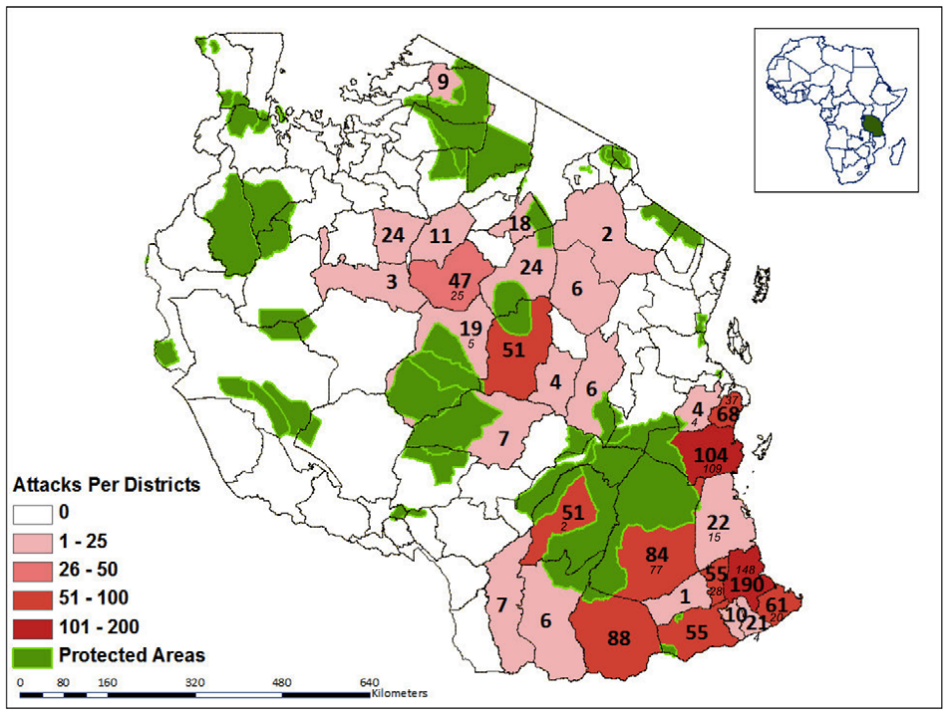
Lion attacks on humans in Tanzania. Bold text shows the number of attack events in each district (1990–2008) [5]; italics indicate the number of victims with known attack times (1988–2009).

**Figure 3:**
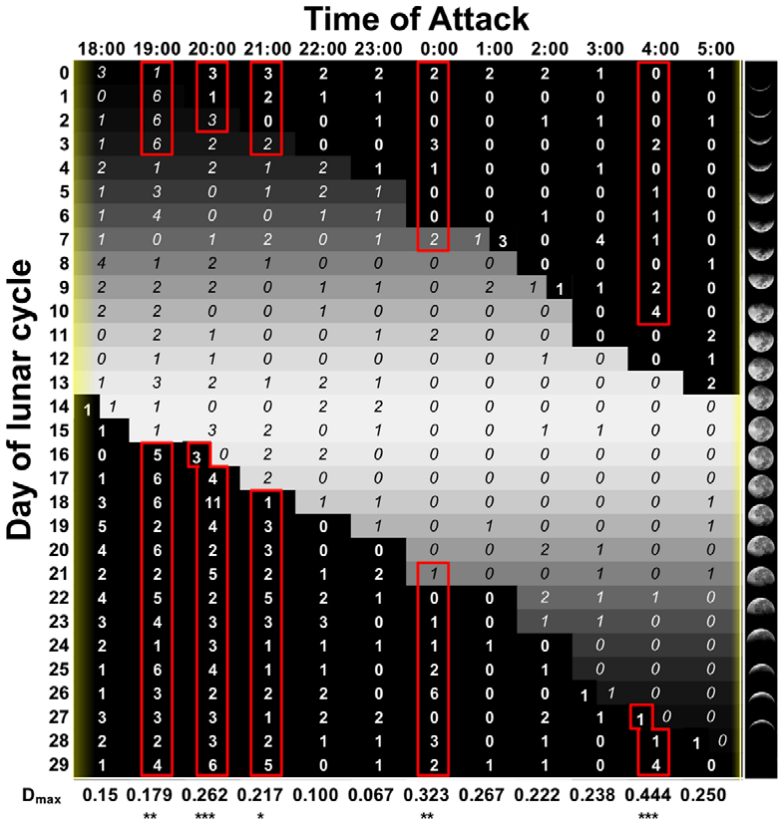
Number of humans attacked by lions each hour across the lunar cycle. Rows indicate the days of the lunar cycle; columns represent time of day between 18:00 and 05:00. Brightness of each cell is proportional to percentage of the moon's illumination. Grey cells with italic font indicate the number of attacks when the moon was above the horizon; black cells with bold font indicate attacks when the moon was below the horizon; several cells have two numbers because of the varying times of sunset/sunrise over the course of the year. Yellow bars indicate dawn (min. 5:55 AM, med. 6:16 AM, max 6:36 AM) and dusk (min. 18:13 PM, med. 18:26 PM, max. 18:48 PM). Red boxes enclose days of the lunar cycle that were significantly over-represented in each hourly distribution according to D_max_ of the Kolmogorov-Smirnov one-sample test (which makes no assumption about the distribution of the data). *P<0.05, **P<0.01, ***P<0.001, ****P<0.0001.

**Figure 4:**
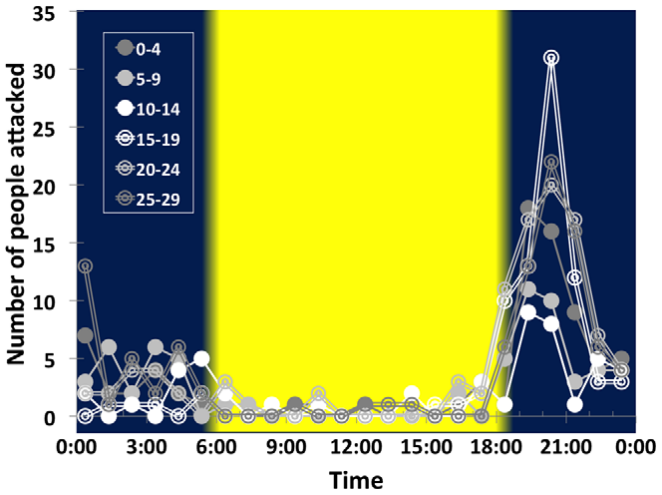
Number of people attacked across 5-day intervals of the lunar cycle. Solid circles: days when the moon was waxing; double circles: days when waning. Yellow area indicates average hours of daylight. Fewer people were attacked between 6:00 and 9:45 PM on the 5^th^–14^th^ days of the lunar cycle (when the moon is increasingly bright and always above the horizon in the evening); most victims were attacked on the 15^th^–29^th^ days (when the moon rises later after sunset each evening).

Lions attacked >1000 Tanzanians between 1988 and 2009 (Fig. 2). Over two-thirds of these attacks were fatal, and the victims were eaten [3], [5]. The vast majority of victims were attacked after dark. Fig. 3 shows the hourly distribution of attacks each night across the lunar cycle: victims were significantly more likely to be attacked during the darkest days of the cycle during five separate hours of night as well as during the darkest parts of the night as a whole. Because lions mostly attack in total darkness and nearly 60% of victims were attacked between 18:00 and 21:45, attack rates varied strikingly with the phase of the moon. Hourly attack rates were 2–4 times higher in the first 10 days after the full moon (when the moon does not rise until after sunset) than in the 10-day period before the full moon (when the moon is 30–100% illuminated and above the horizon at sunset) (Fig. 4).

Moon brightness also varies with weather conditions. Rainfall in southeastern Tanzania is strongly seasonal, and nights are far more likely to be cloudy during the rainy months (Nov.–Dec and Mar.–May) than in the dry season (Jul–Oct.). Season greatly increased the risks of lion attack between 18:00–21:45 during the brightest parts of the lunar cycle. On evenings when the moon was above the horizon and at least 60% illuminated, over three times as many people were attacked each month of the wet season as in the dry season (n = 52 victims, one-sample χ^2^ = 12.05, P<0.001), whereas there was no seasonal difference during evenings when the moon was less than 40% illuminated. A similar analysis on atmospheric attenuation revealed no statistically significant effects of the height of the moon above the horizon.

## Discussion

Lions are nocturnal predators [20], and the overwhelming majority of lion attacks on humans occur at night. Lions are less successful in obtaining wildlife prey during moonlit nights, and moonlight has a similar effect on the risk of human predation. But whereas lions are as likely to feed on herbivore prey during the first and third quarter, most human attacks occur in the weeks following the full moon.

These findings provide novel insights into human attitudes toward the moon. Hominids have always lived in close proximity to large nocturnal carnivores [e.g. 21], [22]. Lions were once the most widely distributed mammal in the world [23]; tigers, jaguars and leopards still co-exist with people in Asia, Africa and tropical America. *Australopithecus* and early *Homo* scavenged from big cats [24]–[25], and *Homo sapiens* painted cave lions with lifelike detail 36,000 yrs ago [26]. Thus we have always been exposed to risks of predation that cycled with the waxing and waning of the moon. Unlike wild herbivores, which remain outdoors throughout the night, people have long slept in shelters [27]. Between sunset and sunrise, humans are most active in the evening [28] and are therefore primarily exposed to predation in the first hours after sunset. The darkest hours in the early evening are restricted to the weeks following the full moon, and lions are hungriest immediately after the bright evenings of the second quarter (Figs. 1a, 3 & 4). Although we are safest from lion attacks during well-lit nights, the *full* moon accurately indicates that the risks of lion predation will increase dramatically in the coming days. Thus the full moon is not dangerous in itself but is instead a portent of the darkness to come.

## Supporting Information

Table S1 Sample sizes for food-intake and carcass-acquisition analyses in the Serengeti and Ngorongoro.
(PDF)

Table S2 Belly size of females in Serengeti and Ngorongoro. Females have lower food intakes during the brightest phase of the moon and in the Serengeti woodlands and plains. Plains females have higher food intake during the wet season.
(PDF)

Table S3 Belly size of male lions in Serengeti and Ngorongoro. Males have lower food intake during the brightest phase of the moon and in the Serengeti plains and woodlands. Plains and woodlands males have higher food intakes during the wet season.
(PDF)

Table S4 Probability of lions being found feeding when first observed each day. Lions were less likely to be found feeding during the brightest phase of the moon and more likely to be found feeding in the Serengeti woodlands and on the Serengeti plains during the wet season.
(PDF)

Table S5 Probability of lions being observed making a kill during the daytime. Daytime kills were more common during the brightest phase of the moon, in the Serengeti woodlands and in the Serengeti plains during the wet season.
(PDF)

Table S6 Probability of lions observed scavenging during the daytime. Scavenging was more common during the brightest phase of the moon and in the Serengeti plains and woodlands, but less common during the wet season in the woodlands.
(PDF)

## References

[1] Kerbis Peterhans JC, Gnoske TP (2001). The science of ‘Man-eating’ among lions (Panthera leo) with a reconstruction of the natural history of the ‘Man-eaters of Tsavo.’. J E Afr Nat Hist.

[2] Löe J, Röskaft E (2004). Large carnivores and human safety: a review.. Ambio.

[3] Packer C, Ikanda D, Kissui B, Kushnir H (2005). Lion attacks on humans in Tanzania.. Nature.

[4] Gurung B, Smith JLD, McDougal C, Karki JB, Barlow A (2008). Factors associated with human-killing tigers in Chitwan National Park, Nepal.. Biol Cons.

[5] Kushnir H, Leitner H, Ikanda D, Packer C (2010). Human and ecological risk factors for unprovoked lion attacks on humans in southeastern Tanzania.. Hum Dimens Wildl.

[6] Van Orsdol KG (1984). Foraging behaviour and hunting success of lions in Queen Elizabeth National Park, Uganda.. Afr J Ecol.

[7] Daly M, Behrends PR, Wilson MI, Jacobsi LF (1992). Behavioural modulation of predation risk: moonlight avoidance and crepuscular compensation in a nocturnal desert rodent, *Dipodomys merriami*.. Anim Behav.

[8] Mougeot F, Bretagnolle V (2000). Predation risk and moonlight avoidance in nocturnal seabirds.. J Avian Biol.

[9] Funston PJ, Mills MGL, Biggs HC (2001). Factors affecting the hunting success of male and female lions in the Kruger National Park.. J Zool.

[10] Di Bitetti MS, Paviolo A, De Angelo C (2006). Density, habitat use and activity patterns of ocelots (*Leopardus pardalis*) in the Atlantic Forest of Misiones, Argentina.. J Zool.

[11] Lang AB, Kalko EKV, Römer H, Bockholdt C, Dechmann DKN (2006). Activity levels of bats and katydids in relation to the lunar cycle.. Oecologia.

[12] Darwin C (1871). The Descent of Man, and Selection in Relation to Sex.

[13] Owens M, McGowan IW (1998). Madness and the moon: the lunar cycle and psychopathology.. Ger J Psychiatr.

[14] Voracek M, Loibl LM, Kapusta ND, Niederkrotenthaler T, Dervic K (2008). Not carried away by a moonlight shadow: no evidence for associations between suicide occurrence and lunar phase among more than 65,000 suicide cases in Austria, 1970–2006.. Wien Klin Wochenschr.

[15] Foster RG, Roenneberg T (2008). Human responses to the geophysical daily, annual and lunar cycles.. Curr Biol.

[16] Kissui BM, Packer C (2004). Top-down regulation of a top predator: lions in the Ngorongoro Crater.. Proc Roy Soc Series B.

[17] Packer C, Hilborn R, Mosser A, Kissui B, Wilmshurst J (2005). Ecological change, group territoriality and non-linear population dynamics in Serengeti lions.. Science.

[18] Bertram BCR (1975). Weights and measures of lions.. E Afr Wildl J.

[19] R Development Core Team (2009). R: a language and environment for statistical computing..

[20] Hayward MW, Slotow R (2009). Temporal partitioning of activity in large African carnivores: Tests of multiple hypotheses.. S Afr J Wildl Res.

[21] Gilbert WH, Asfaw B (2008). Homo erectus: Pleistocene evidence from the Middle Awash, Ethiopia.

[22] Lewis ME, Werdelin L, Bobe R, Alemseged Z, Behrensmeyer AK (2007). Patterns of change in the Plio-Pleistocene carnivorans of eastern Africa. Implications for hominin evolution.. Hominin Environments in the East African Pliocene: An Assessment of the Faunal Evidence.

[23] Antunes A, Troyer JL, Roelke ME, Pecon-Slattery J, Packer C (2008). The evolutionary history of lion: Integrating host/pathogen molecular genetics.. PLoS Genet.

[24] de Heinzelin J, Clark JD, White T, Hart W, Renne P (1999). Environment and behavior of 2.5-million-year-old Bouri hominids.. Science.

[25] McPherron SP, Alemseged Z, Marean CW, Wynn JG, Reed D (2010). Evidence for stone-tool-assisted consumption of animal tissues before 3.39 million years ago at Dikika, Ethiopia.. Nature.

[26] Clottes J, Packer C, Clottes J (2001). Les félins.. La Grotte Chauvet: L'Art des Origines.

[27] Klein R (2009). The Human Career, 3^rd^ Edn.

[28] Worthman CM, Melby MK, Carskadon MA (2002). Toward a comparative developmental ecology of human sleep.. Adolescent Sleep Patterns: Biological, Social, and Psychological Influences.

