# Supplementary material for: Fear of Darkness, the Full Moon and the Nocturnal Ecology of African Lions
Source: PLoS One. 2011 Jul 20;6(7):e22285. doi: 10.1371/journal.pone.0022285 (PMC3140494; doi:10.1371/journal.pone.0022285)
Supplement: Table S1 — Sample sizes for food-intake and carcass-acquisition analyses in the Serengeti and Ngorongoro. (PDF) [file pone.0022285.s001.pdf]

Table S1. Sample sizes for food-intake and carcass-acquisition analyses in the Serengeti and Ngorongoro.

Data for belly size regression models

	**N *females***	**N *males***	**Years of data**
**Ngorongoro Crater**	1584	1154	26
**Serengeti Plains**	2867	1256	29
**Serengeti Woodlands**	3232	1417	29
**total**	***7,683***	***3,827***	

N = number of sightings of known-aged lions where belly size was recorded, taking only 1 sighting per pride per day

Data for carcass regressions

**Habitat**	**Kills**	**Scavenge**	**Previously obtained**	**Total Observations**
**Ngorongoro Crater**	291	42	486	7060
**Serengeti Plains**	379	113	842	16353
**Serengeti Woodlands**	607	120	1454	21212
**Serengeti nomads**	48	20	193	1448
**total**	***1325***	***295***	***2975***	***46073***

N = number of sightings since 1966 in Serengeti and 1975 in Ngorongoro.
"Previously obtained" - lions were found feeding when first observed that day
