# Supplementary material for: Fear of Darkness, the Full Moon and the Nocturnal Ecology of African Lions
Source: PLoS One. 2011 Jul 20;6(7):e22285. doi: 10.1371/journal.pone.0022285 (PMC3140494; doi:10.1371/journal.pone.0022285)
Supplement: Table S2 — Belly size of females in Serengeti and Ngorongoro. Females have lower food intakes during the brightest phase of the moon and in the Serengeti woodlands and plains. Plains females have higher food intake during the wet season. (PDF) [file pone.0022285.s002.pdf]

Table S2. Belly size of females in Serengeti and Ngorongoro. Females have lower food intakes during the brightest phase of the moon and in the Serengeti woodlands and plains. Plains females have higher food intake during the wet season.

Female belly size: Model Summary

Linear mixed model fit by maximum likelihood

*Formula: avg.belly ~ luminosity + habitat + season + habitat*season + (1|pride) + (1|year)*

Random effects:

**Groups**	**Name**	**Variance**	**Std.Dev.**
Pride	(Intercept)	0.0026491	0.051469
Year	(Intercept)	0.0205052	0.143196
Residual		0.3067	0.5538

Number of observations: 7683, groups: pride, 68; year, 29

Fixed effects:

	**Estimate**	**Std. Error**	**t value**
(Intercept)	2.50789	0.03947	63.54
Luminosity	-0.1102	0.01800	-6.13
PlainsHabitat	-0.2110	0.03424	-6.16
WoodlandsHabitat	-0.1043	0.0341	-3.06
WetSeason	-0.0102	0.0285	-0.36
Plains:WetSeason	0.2511	0.0355	7.08
Woodlands:WetSeason	-0.0165	0.0350	-0.47

Females: ANOVA Comparison of final model to the next simplest model

♀ *model w/o luminosity: avg.belly ~ habitat+season+habitat*season+(1|pride)+(1|year)*

♀ *model w/luminosity: avg.belly ~ luminosity+habitat+season+habitat*season+(1|pride)+(1|year)*

	**Df**	**AIC**	**BIC**	**logLik**	χ^2	**Df**	**Pr(>χ^2)**
**Females w/o luminosity**	9	12896.2	12958.8	-6439.1			
**Females w/luminosity**	10	12860.8	12930.2	-6420.4	37.5	1	9.21E-10
