# Supplementary material for: Fear of Darkness, the Full Moon and the Nocturnal Ecology of African Lions
Source: PLoS One. 2011 Jul 20;6(7):e22285. doi: 10.1371/journal.pone.0022285 (PMC3140494; doi:10.1371/journal.pone.0022285)
Supplement: Table S3 — Belly size of male lions in Serengeti and Ngorongoro. Males have lower food intake during the brightest phase of the moon and in the Serengeti plains and woodlands. Plains and woodlands males have higher food intakes during the wet season. (PDF) [file pone.0022285.s003.pdf]

Table S3. Belly size of male lions in Serengeti and Ngorongoro. Males have lower food intake during the brightest phase of the moon and in the Serengeti plains and woodlands. Plains and woodlands males have higher food intakes during the wet season.

Male belly size: Model Summary

Linear mixed model fit by maximum likelihood

*Formula: avgbelly ~ luminosity + habitat + season + habitat*season + (1|pride) + (1|year)*

Random effects:

Groups	Name	Variance	Std.Dev.
Pride	(Intercept)	0.0068098	0.082522
Year	(Intercept)	0.0270298	0.164407
Residual		0.3754961	0.612777

Number of observations: 3827, groups: pride, 63; year, 29

Fixed effects:

	Estimate	Std. Error	t value
(Intercept)	2.31907	0.05391	43.02
Luminosity	-0.11262	0.02823	-3.99
PlainsHabitat	-0.37533	0.05599	-6.70
WoodlandsHabitat	-0.17724	0.05487	-3.23
WetSeason	-0.09045	0.03735	-2.42
Plains:WetSeason	0.39960	0.05267	7.59
Woodlands:WetSeason	0.15616	0.05036	3.10

Male belly size: ANOVA Comparison of final model to the next simplest model

♂ *model w/o luminosity: avg.belly ~ habitat+season+habitat*season+(1|pride)+(1|year)*

♂ *model w/luminosity: avg.belly ~ luminosity+habitat+season+habitat*season+(1|pride)+(1|year)*

	Df	AIC	BIC	logLik	χ^2	Df	Pr(>χ^2)
**Males w/o luminosity**	9	7244.5	7300.8	-3613.3			
**Males w/luminosity**	10	7230.7	7293.2	-3605.3	15.871	1	6.78E-05
