# Supplementary material for: Fear of Darkness, the Full Moon and the Nocturnal Ecology of African Lions
Source: PLoS One. 2011 Jul 20;6(7):e22285. doi: 10.1371/journal.pone.0022285 (PMC3140494; doi:10.1371/journal.pone.0022285)
Supplement: Table S4 — Probability of lions being found feeding when first observed each day. Lions were less likely to be found feeding during the brightest phase of the moon and more likely to be found feeding in the Serengeti woodlands and on the Serengeti plains during the wet season. (PDF) [file pone.0022285.s004.pdf]

Table S4. Probability of lions being found feeding when first observed each day. Lions were less likely to be found feeding during the brightest phase of the moon and more likely to be found feeding in the Serengeti woodlands and on the Serengeti plains during the wet season.

PREVIOUSLY OBTAINED CARCASSES

Call: glm(formula = count ~ luminosity + habitat*season, family = Poisson)

Deviance Residuals:

Min	1Q	Median	3Q	Max
-2.8107	-0.8725	-0.2384	0.6873	2.6058

Coefficients:

	Estimate	Std. Error	z value	Pr(>|z|)
(Intercept)	2.84131	0.06950	40.884	< 2e-16
Luminosity	-0.14104	0.05382	-2.620	0.00878
PlainsHabitat	0.13249	0.08841	1.499	0.13398
WoodsHabitat	1.10277	0.07450	14.803	< 2e-16
WetSeason	0.02469	0.09073	0.272	0.78550
Plains:WetSeason	0.70430	0.11680	6.030	1.64E-09
Woods:WetSeason	-0.01369	0.10480	-0.131	0.89600

(Dispersion parameter for Poisson family taken to be 1)
Null deviance: 761.48 on 89 degrees of freedom
Residual deviance: 132.31 on 83 degrees of freedom
AIC: 607.51
Number of Fisher Scoring iterations: 4

Analysis of Deviance Table

Model 1: count ~ habitat * season
Model 2: count ~ luminosity + habitat * season

	Resid. Df	Resid. Dev	Df	Deviance	$P(>\|\chi^2\|)$
**w/o luminosity**	84	139.178			
**w/ luminosity**	83	132.306	1	6.872	0.008754
