# Supplementary material for: Fear of Darkness, the Full Moon and the Nocturnal Ecology of African Lions
Source: PLoS One. 2011 Jul 20;6(7):e22285. doi: 10.1371/journal.pone.0022285 (PMC3140494; doi:10.1371/journal.pone.0022285)
Supplement: Table S5 — Probability of lions being observed making a kill during the daytime. Daytime kills were more common during the brightest phase of the moon, in the Serengeti woodlands and in the Serengeti plains during the wet season. (PDF) [file pone.0022285.s005.pdf]

Table S5. Probability of lions being observed making a kill during the daytime. Daytime kills were more common during the brightest phase of the moon, in the Serengeti woodlands and in the Serengeti plains during the wet season.

DAYTIME KILLS

Call: glm(formula = count ~ luminosity + habitat*season, family = Poisson)

Deviance Residuals:

Min	1Q	Median	3Q	Max
-2.5495	-0.7235	-0.1364	0.7352	3.0861

Coefficients:

	Estimate	Std. Error	z value	Pr(>|z|)
(Intercept)	2.15443	0.09097	23.684	< 2e-16
Luminosity	0.36144	0.07986	4.526	6.01E-06
PlainsHabitat	0.09764	0.11056	0.883	0.377
WoodsHabitat	0.74620	0.09721	7.676	1.64E-14
WetSeason	-0.14458	0.11755	-1.230	0.219
Plains:WetSeason	0.32981	0.15641	2.109	0.035
Woods:WetSeason	-0.02386	0.14302	-0.167	0.868

(Dispersion parameter for Poisson family taken to be 1)
Null deviance: 260.25 on 89 degrees of freedom
Residual deviance: 109.15 on 83 degrees of freedom
AIC: 519.20
Number of Fisher Scoring iterations: 4

Analysis of Deviance Table

Model 1: count ~ habitat * season
Model 2: count ~ luminosity + habitat * season

	Resid.Df	Resid. Dev	Df	Deviance	$P(>\|\chi^2\|)$
**w/o luminosity**	84	129.76			
**w/ luminosity**	83	109.15	1	20.61	5.63E-06
