# Supplementary material for: Fear of Darkness, the Full Moon and the Nocturnal Ecology of African Lions
Source: PLoS One. 2011 Jul 20;6(7):e22285. doi: 10.1371/journal.pone.0022285 (PMC3140494; doi:10.1371/journal.pone.0022285)
Supplement: Table S6 — Probability of lions observed scavenging during the daytime. Scavenging was more common during the brightest phase of the moon and in the Serengeti plains and woodlands, but less common during the wet season in the woodlands. (PDF) [file pone.0022285.s006.pdf]

Table S6. Probability of lions observed scavenging during the daytime. Scavenging was more common during the brightest phase of the moon and in the Serengeti plains and woodlands, but less common during the wet season in the woodlands.

SCAVENGING

Call: glm(formula = count ~ luminosity + habitat*season, family = Poisson)

Deviance Residuals:

Min	1Q	Median	3Q	Max
-2.8081	-0.9708	-0.2116	0.6237	2.5691

Coefficients:

	Estimate	Std. Error	z value	Pr(>|z|)
(Intercept)	-0.5140	0.2871	-1.79	7.34E-02
Luminosity	0.8148	0.1763	4.62	3.83E-06
PlainsHabitat	1.1896	0.3052	3.898	9.72E-05
WoodsHabitat	1.7304	0.2900	5.968	2.41E-09
WetSeason	0.6931	0.3273	2.118	0.034199
Plains:WetSeason	-0.3171	0.3792	-0.836	0.403034
Woods:WetSeason	-1.3490	0.3797	-3.553	0.000381

(Dispersion parameter for Poisson family taken to be 1)
Null deviance: 197.64 on 89 degrees of freedom
Residual deviance: 08.34 on 83 degrees of freedom
AIC: 352.89
Number of Fisher Scoring iterations: 5

Analysis of Deviance Table

Model 1: count ~ habitat * season
Model 2: count ~ luminosity + habitat * season

	Resid. Df	Resid. Dev	Df	Deviance	$P(>\|\chi^2\|)$
**w/o luminosity**	84	130.347			
**w/ luminosity**	83	108.338	1	22.009	2.71E-06
